# Coated and Hollow Microneedle-Mediated Intradermal Immunization in Mice with Diphtheria Toxoid Loaded Mesoporous Silica Nanoparticles

**DOI:** 10.1007/s11095-018-2476-4

**Published:** 2018-08-13

**Authors:** Guangsheng Du, Laura Woythe, Koen van der Maaden, Mara Leone, Stefan Romeijn, Alexander Kros, Gideon Kersten, Wim Jiskoot, Joke A. Bouwstra

**Affiliations:** 1 Division of BioTherapeutics, Leiden Academic Centre for Drug Research, Leiden University, Leiden, The Netherlands; 2 Department of Supramolecular & Biomaterials Chemistry, Leiden Institute of Chemistry, Leiden University, Leiden, The Netherlands; 3 Institute for Translational Vaccinology (Intravacc), Bilthoven, The Netherlands

**Keywords:** coated microneedles, diphtheria toxoid, hollow microneedles, intradermal vaccination, mesoporous silica nanoparticles

## Abstract

**Purpose:**

To examine the immunogenicity of diphtheria toxoid (DT) loaded mesoporous silica nanoparticles (MSNs) after coated and hollow microneedle-mediated intradermal immunization in mice.

**Methods:**

DT was loaded into MSNs and the nanoparticle surface was coated with a lipid bilayer (LB-MSN-DT). To prepare coated microneedles, alternating layers of negatively charged LB-MSN-DT and positively charged N-trimethyl chitosan (TMC) were coated onto pH-sensitive microneedle arrays via a layer-by-layer approach. Microneedle arrays coated with 5 or 3 layers of LB-MSN-DT were used to immunize mice and the elicited antibody responses were compared with those induced by hollow microneedle-injected liquid formulation of LB-MSN-DT. Liquid DT formulation with and without TMC (DT/TMC) injected by a hollow microneedle were used as controls.

**Results:**

LB-MSN-DT had an average size of about 670 nm and a zeta potential of −35 mV. The encapsulation efficiency of DT in the nanoparticles was 77%. The amount of nano-encapsulated DT coated onto the microneedle array increased linearly with increasing number of the coating layers. Nano-encapsulated DT induced stronger immune responses than DT solution when delivered intradermally via hollow microneedles, but not when delivered via coated microneedles.

**Conclusion:**

Both the nano-encapsulation of DT and the type of microneedles affect the immunogenicity of the antigen.

## Introduction

Vaccination is one of the most cost-effective tools to prevent infectious diseases in human beings (1). Traditional vaccines are based on attenuated or inactivated pathogens. Nowadays, subunit vaccines containing only immunogenic parts of a pathogen are being extensively investigated because they are safer (2). The disadvantage of subunit vaccines is that they are generally less immunogenic than traditional vaccines. To overcome this, adjuvants such as immune modulators and nanoparticulate delivery systems can be used (3,4).

Nanoparticles have been extensively studied for the delivery of vaccines, as they can improve the immunogenicity of antigens by enhancing the targeting of antigens to antigen-presenting cells (APCs) (5). Furthermore, the immune responses can potentially be modified by tuning the properties of nanoparticles such as size, surface charge, and release kinetics of antigens (3,6,7). Among different types of nanoparticles, mesoporous silica nanoparticles (MSNs) have gained increasing attention because of their excellent biocompatibility and stability. Besides, the silica surface can be easily modified and functionalized and the large pores and surface area of MSNs enable efficient loading of antigens with a high loading capacity (8,9). Studies have shown that antigen loaded MSNs are able to increase the uptake of antigens by APCs and improve immune responses in mice (9–11).

Vaccines are mostly administered by intramuscular or subcutaneous injection, but these methods have disadvantages such as low acceptance by a considerable number of people and infection risk due to needlestick injuries or reuse of needles (12–14). Additionally, the delivery of vaccines to APCs may be inefficient as these delivery sites are not rich of APCs (15). To avoid the drawbacks of hypodermic needles, microneedles have been developed. Microneedles are micrometer-sized needle-like structures and can be used to penetrate skin and deliver the antigen in a minimal invasive and pain-free way (16). The skin contains a large number of APCs, and therefore microneedle-mediated intradermal delivery of vaccines has potential for effective vaccination (17).

Several types of microneedles are in development, such as coated, dissolvable and hollow microneedles (16). On the one hand, coated and dissolvable microneedles are used to administer dry-state vaccine formulations (18), which offer the potential advantage of improving antigen stability (16,19). Previously, silicon microneedle arrays with a pH-sensitive surface were developed to bind negatively charged vaccines at slightly acidic conditions (pH 5.8) and release the coated material at physiological pH (7.4) (20). Several studies have shown that the antigen coated microneedles induced a similar immune response as subcutaneously or intramuscularly injected antigen solution (21–23). On the other hand, hollow microneedles are used to inject liquid formulations and the dose can be precisely controlled. We previously showed that hollow microneedles together with an applicator can be used to deliver antigen-loaded nanoparticles intradermally (24).

In this study, we aimed to examine the immunogenicity of intradermally delivered DT loaded MSNs by using either coated microneedle arrays or a single hollow microneedle. The microneedle arrays were coated with DT loaded in MSNs by using a layer-by-layer coating approach after which the delivered dose into *ex vivo* human skin was examined. In a subsequent immunization study, the antibody response induced by LB-MSN-DT coated microneedles was compared with that obtained after injection of a suspension of LB-MSN-DT by hollow microneedles into mouse skin.

## Materials and Methods

### Materials

DT (batch 04–44, 1 μg equal to 0.3 Lf) and diphtheria toxin were provided by Intravacc (Bilthoven, The Netherlands). (3-aminopropyl)triethoxysilane (APTES, 99%), 4-pyridinecarboxaldehyde (97%), sodium cyanoborohydride (NaBH_3_CN, 95%), cholesterol (≥99%), fetal bovine serum (FBS), M199 medium (with Hank’s salts and L-glutamine) and bovine serum albumin (BSA) were obtained from Sigma-Aldrich (Zwijndrecht, The Netherlands). 1,2-dioleoyl-sn-glycero-3-phosphocholine (DOPC) and 1,2-dioleoyl-sn-glycero-3-[phospho-L-serine](sodium salt) (DOPS) were purchased from Avanti Polar Lipids Inc. (Alabaster, AL). Hydrogen peroxide (30%) was purchased from Fluka (Steinheim, Germany). Toluene (≥99.7%) was obtained from Biosolve (Valkenswaard, The Netherlands). N-trimethyl chitosan (TMC) and rhodamine labeled TMC (TMC-Rho) were prepared as reported previously (23,25). Glucose solution, L-glutamine (200 nM), penicillin-streptomycin (10,000 U/mL) and 1-step™ ultra 3,3′,5,5′-tetramethylbenzidine (TMB) were purchased from Thermo-Fisher Scientific (Waltham, MA). IRDye 800CW protein labeling kit (low molecular weight) was ordered from LI-COR (Lincoln, NE). HRP-conjugated goat anti-mouse total IgG, IgG1 and IgG2a were ordered from Southern Biotech (Birmingham, AL). Sulfuric acid (95–98%) was obtained from JT Baker (Deventer, The Netherlands). Sterile phosphate buffered saline (PBS, 163.9 mM Na^+^, 140.3 mM Cl^−^, 8.7 mM HPO_4_^2−^, 1.8 mM H_2_PO^4−^, pH 7.4) was ordered from B. Braun (Oss, The Netherlands). 1 mM phosphate buffer (PB) with a pH of 7.4 or 5.8 was prepared in the lab. Milli-Q water (18 MΩ/cm, Millipore Co.) was used for the preparation of all solutions. All the other chemicals used were of analytical grade.

### Preparation of DT Encapsulated and Lipid Fused MSNs (LB-MSN-DT)

Plain MSNs with a particle size of about 200 nm and large pores (about 10 nm in diameter) were prepared and modified with amino groups to generate a positively charged surface, as described earlier (11,26). To improve the colloidal stability of MSNs, liposomes were coated onto the surface of MSNs by using a method as previously described (11). These liposomes were prepared by lipid film hydration followed by sonication. Briefly, DOPC, DOPS and cholesterol with a molar ratio of 7:1:2 were dissolved in chloroform in a round bottom flask. The organic solvent was evaporated by using a rotary evaporator (Buchi rotavapor R210, Flawil, Switzerland) for 30 min. Subsequently, the lipid film was hydrated with 1 mM PB (pH 7.4) and vortexed for 10 s to form a lipid vesicle suspension. The suspension was sonicated in a Branson 2510 water bath (Danbury, CT) for 10 min. The obtained liposomes were stored at 4°C in the refrigerator for further use.

To prepare LB-MSN-DT, 0.5 mL MSNs (2 mg/mL) and 0.5 mL DT (0.5 mg/mL) were mixed in 1 mM PB (pH 7.4), followed by addition of 0.5 mL liposomes (2 mg/mL) in 1 mM PB (pH 7.4). To prepare LB-MSN-DT loaded with Alexa488 or IRDye 800CW labeled DT, plain DT was replaced with fluorescently labeled DT according the need of experiments. The mixture was incubated in an Eppendorf thermomixer (Nijmegen, The Netherlands) for 1.5 h at 25°C with a speed of 300 rpm. To remove the excess DT and liposomes, the suspension was centrifuged by using a Sigma 1–15 centrifuge (Osterode, Germany) for 5 min with a speed of 10,000 g. The resultant pellet was washed and re-dispersed in 1 mM PB (pH 7.4) for further use.

### Measurement of Size and Zeta Potential of LB-MSN-DT

The size and zeta potential of LB-MSN-DT were determined by using dynamic light scattering (DLS) and laser Doppler velocimetry, respectively, with a Nano ZS® zetasizer (Malvern Instruments, Worcestershire, U.K.). The samples were diluted in 1 mM PB (pH 7.4) to a concentration of 25 μg/mL (expressed based on the concentration of MSNs) and measured 3 times with 10 runs for each measurement.

### Determination of Encapsulation Efficiency (EE) and Loading Capacity (LC) of DT in LB-MSN-DT

The loading efficiency of DT was determined by measuring the intrinsic fluorescence intensity of DT ((λ_ex_ 280 nm/λ_em_ 320 nm) in the supernatant before and after encapsulation by using a Tecan M1000 plate reader (Männedorf, Switzerland). The EE and LC were calculated using the equations below:

$$ \mathrm{EE}=\frac{M_{loaded\  DT}\ }{M_{total\  DT}}\times 100\% $$

$$ \mathrm{LC}=\frac{M_{loaded\  DT}\ }{M_{MSNs}}\times 100\% $$

Where *M*_*loaded DT*_ represents the mass of encapsulated DT, *M*_*total DT*_ is the total amount of DT added to the formulation and *M*_*MSNs*_ is the weight of MSNs.

### *In Vitro* Release of DT from LB-MSN-DT

To study the release of DT, 1 mL nanoparticle suspension with a concentration of 1 mg/mL (expressed based on the concentration of MSNs, corresponding to about 0.2 mg/mL DT) in PBS was incubated for one month at 37°C by using an Eppendorf thermomixer (Nijmegen, The Netherlands) set at a speed of 550 rpm. At predetermined time points, the samples were centrifuged for 5 min with a speed of 10,000 g. 600 μL sample from the supernatant was collected and the amount of DT was measured by intrinsic fluorescence intensity of DT. Fresh PBS with the same volume of the collected supernatant was added back to the suspension. The release percentage of DT was calculated by dividing the released amount of DT by the total amount of DT initially loaded in LB-MSN-DT.

### Modification of Microneedle Arrays to Achieve a PH-Sensitive Surface

Silicon microneedle arrays with 576 microneedles per array on a back plate of 5 × 5 mm^2^ with a microneedle length of 200 μm were kindly provided by Robert Bosch GmbH (Stuttgart, Germany). To obtain pH-sensitive microneedles, the surface was modified with pyridine groups as previously reported (20). In brief, the microneedle surface was first cleaned by piranha solution (70% sulfuric acid and 30% hydrogen peroxide) at 120°C for 2 h. *Caution: piranha is strongly acidic and oxidizing. Piranha reacts violently with organic compounds, and it should not be stored in closed containers.* Subsequently, the microneedles were extensively washed with MilliQ water followed by washing with acetone and methanol. Next, the microneedles were incubated in 2% APTES in toluene overnight to obtain an amine-modified surface and thereafter incubated with 4-pyridinecarboxaldehyde (100 mM) in anhydrous isopropanol containing 1% acetic acid overnight. Finally, the formed imine bonds were reduced to secondary amines by incubating the microneedles with NaBH_3_CN (50 mM) in isopropanol for 2 h. After cleaning the microneedles were stored under vacuum at 50°C until further use.

### Multilayer Coating of LB-MSN-DT on the Surface of Microneedle Arrays

LB-MSN-DT and TMC were alternately coated onto the surface of microneedle arrays by using a layer-by-layer approach. The pH-sensitive microneedle arrays were transferred into Greiner Cellstar® 48 well plates. 50 μL negatively charged LB-MSN-DT (0.5 mg/mL) in 1 mM PB (pH 5.8) was added onto the top of each microneedle array and the arrays were incubated for 30 min. The excess nanoparticles were washed by adding 450 μL 1 mM PB (pH 5.8). Next, the microneedle arrays were dried under pressurized nitrogen flow for 10 min. After the first coating layer of LB-MSN-DT, 50 μL positively charged TMC (40 μg/mL) in 1 mM PB (pH 5.8) was added onto the top of each microneedle array and the arrays were incubated with TMC for another 30 min. The concentration of LB-MSN-DT and TMC in the coating solutions were chosen based on prior studies (11,23). The excess TMC was removed by washing the microneedle arrays with 450 μL 1 mM PB (pH 5.8). Subsequently, the microneedle arrays were dried under nitrogen flow as described above. This procedure was repeated until the desired number of coating layers of LB-MSN-DT was reached. After the last layer of LB-MSN-DT, no more TMC was coated onto the microneedle surface. In order to study the dose effect of DT using coated microneedles, the microneedle arrays were coated with either 5 or 3 layers of LB-MSN-DT and 4 or 2 alternate layers of TMC, respectively.

To determine the coating efficiency of DT on microneedles, the amount of nano-encapsulated DT in the supernatant after washing was determined by measuring the intrinsic fluorescence of DT. The coating efficiency was calculated by dividing the amount of coated DT by the total amount of DT initially added to the coating solution.

### Insertion of Microneedle Arrays into *Ex Vivo* Human Skin

*Ex vivo* human skin was obtained from a local hospital according to Helsinki principles. A written informed patient consent was obtained. To reproducibly insert the microneedles into the skin, an in-house developed impact-insertion injector together with a uPRAX applicator controller (Delft, The Netherlands) was used by using either a single insertion mode or multiple insertion mode (27,28). In case of a single insertion, the microneedle arrays were inserted into the skin with an average velocity of 0.5 m/s and kept in the skin for 30 min by applying a force of 5 N on top of the microneedle array. In case of multiple insertion mode, the microneedle arrays were 10 times inserted into the skin within 10 s with an average velocity of 0.5 m/s. After the last penetration, the microneedles were removed from the skin.

### Visualization of the Coated Microneedles before and after Penetration of *Ex Vivo* Human Skin by Scanning Electronic Microscopy (SEM)

The 5-layer LB-MSN-DT coated microneedles were visualized with a Nova NanoSEM (Eindhoven, The Netherlands) operated with a voltage of 15 kV before and after removal from the skin. To increase the surface conductivity, the microneedle arrays were coated with a layer of platina/palladium before visualization.

### Release of LB-MSN-DT from Microneedle Arrays into *Ex Vivo* Human Skin

To visualize the release of LB-MSN-DT from microneedle arrays into the skin, the 5-layer LB-MSN-DT coated microneedle arrays and the released nanoparticles in the skin were visualized by using a Nikon D-Eclipse C1 CLSM (Tokyo, Japan). For this purpose, DT-Alexa488 and TMC-Rho were used. The coated microneedles and the skin area penetrated by coated microneedles were scanned with a depth resolution of 5 μm/step by using a 10 × and 4 × Plan Apo objective, respectively. An argon laser (488 nm) with a 530/55 emission filter and a diode-pumped solid-state laser (561 nm) with a 590/55 emission filter were used for visualization of DT-Alexa488 and TMC-Rho, respectively.

The released amount of DT in the *ex vivo* human skin was quantified by using a Perkin-Elmer IVIS Lumina Series III *in vivo* imaging system (Waltham, MA, USA). For this purpose, DT was labeled with IRDye 800 CW (DT-IRDye800) by using a IRDye 800CW protein labeling kit (low molecular weight) according to the manufacturer’s instruction. The LB-MSN-DT-IRDye800 coated microneedles were inserted into human skin by using either the single or multiple insertion mode as described above. A calibration curve was prepared by injecting different amounts of LB-MSN-DT-IRDye800 in the skin by using a hollow microneedle (see below). To determine the amount of DT released from the coated microneedles, the fluorescence intensity of DT-IRDye800 in the skin was measured by using the *in vivo* imaging system with a 745 nm excitation wavelength and an ICG emission filter. By using the calibration curve the amount of delivered DT was calculated.

### Hollow Microneedles and Applicator

The hollow microneedles were prepared by etching of fused silica capillaries with hydrofluoric acid, as previously described (29). In brief, silica capillaries (375 μm outer diameter, 50 μm inner diameter) were cut into 4-cm pieces and filled with silicone oil in a vacuum oven (100°C) overnight. The tips of capillaries were etched in ≥48% hydrofluoric acid for 4 h. Subsequently, the polyimide coating was removed by dipping the microneedle tips into hot sulfuric acid (250°C) for 5 min. The applicator for hollow microneedles consists of a syringe pump and an injector for precise control of injection depth, rate and volume. The hollow microneedles, injector and pump were connected by silica capillaries and high-pressure resistant CapTite™ connectors (24).

### Immunization Studies in Mice

Female BALB/c mice of 7–8 weeks old (Charles River, Maastricht, The Netherlands) at the start of the experiments were used for the immunization study. The animals were housed under standardized conditions in the animal facility of Leiden Academic Centre for Drug Research. The study was approved by the ethical committee on animal experiments of Leiden University (Licence number 14166).

Mice were first anesthetized by intraperitoneal injection of ketamine (60 mg/kg) and xylanize (4 mg/kg) before shaving the abdomen area. In case of coated microneedles, the LB-MSN-DT coated microneedle arrays were inserted into the abdomen of mice by using the multiple insertion mode as described above for the studies in *ex vivo* human skin. Each mouse was immunized with one microneedle array coated with either 5 or 3 layers of LB-MSN-DT. In case of hollow microneedles, the following groups were included: a) 10 μL suspension of LB-MSN-DT, b) 10 μl DT solution and c) 10 μl DT and TMC solution. All formulations of hollow microneedle groups contained 0.31 μg DT. The same amount of TMC was included in the DT and TMC group. The formulation was injected into the skin of the abdomen of mice with a rate of 10 μL/min at a depth of 120 μm. Subcutaneously injected 5 μg DT formulated with 150 μg colloidal aluminum phosphate (DT-Alum) in PBS with a volume of 100 μL was used as a positive control. The mice were immunized on day 0 (prime), 21 (1st boost), 42 (2nd boost) and sacrificed on day 56. The serum was withdrawn from the tail veins of the mice on day 0, 21 and 42 prior to the immunization. On day 56 the serum was collected from femoral vein and the mice were sacrificed by cervical dislocation.

### Measurement of DT-Specific Antibody Titers

The total IgG and subtype IgG1 and IgG2a titers in the serum were measured by using ELISA as previously reported (30). Briefly, the wells of 96-well plates were first coated with 140 ng DT overnight at 4°C. Next, the plates were blocked with 1% BSA and appropriate 3-fold serial diluted serum samples were applied to the plates and incubated for 2 h at 37°C. Subsequently, HRP-conjugated goat anti-mouse total IgG, IgG1 and IgG2a were added into the wells and incubated for 1.5 h. Finally, TMB was added to the plates and 2 M sulfuric acid was added to stop the reaction. The absorbance was measured at 450 nm by using a Tecan M1000 plate reader. The antibody titers were expressed as the ^10^log value where the corresponding absorbance is located in the middle of the S-shaped dilution-absorbance curve.

### Measurement of DT-Neutralizing Antibody Titers

To check the functionality of the antibodies, diphtheria toxin neutralizing antibody titers in the serum of the mice at day 56 were checked by using a Vero-cell assay (31). Briefly, appropriate 2-fold serial diluted serum was first applied to 96-well plates. 5 × 10^−5^ Lf diphtheria toxin was added to each well and incubated for 2 h at 37°C in a stove with 5% CO_2_. Subsequently, 1.25 × 10^4^ Vero cells were added to each well and incubated for 6 days at 37°C in the stove with 5% CO_2._ Finally, the neutralizing antibodies were shown as the ^2^log value of the highest dilution times of serum that protected the Vero cells.

### Statistics Analysis

All the data of antibody titers were analyzed by one way ANOVA with Newman-Keuls Multiple post-test by using GraphPad Prism software (version 5.02). The level of significance was set at *p < 0.05, **p < 0.01, ***p < 0.001.

## Results

### Physicochemical Characteristics of LB-MSN-DT

The physicochemical characteristics of LB-MSN-DT are shown in Table I. The size of LB-MSN-DT was approximately 700 nm with a polydispersity index (PDI) slightly larger than 0.3. The nanoparticles showed a high negative zeta potential. DT was efficiently encapsulated into the nanoparticles with a high EE and LC.

**Table I:** Physicochemical Characteristics of LB-MSN-DT (*n* = 3).

Nanoparticles	Size^a^ (nm)	PDI^b^	ZP^c^ (mV)	EE%^d^	LC%^e^
LB-MSN-DT	676 ± 7	0.322 ± 0.016	−35 ± 1	77.1 ± 6.4	19.3 ± 1.6

Data are average ± SEM of 3 independent batches

^a^Size: Z-average in diameter, ^b^PDI: polydispersity index, ^**c**^ZP: zeta potential, ^**d**^EE: encapsulation efficiency, ^**e**^LC: loading capacity

### *In Vitro* Release of DT from LB-MSN-DT

**Fig. 1:**
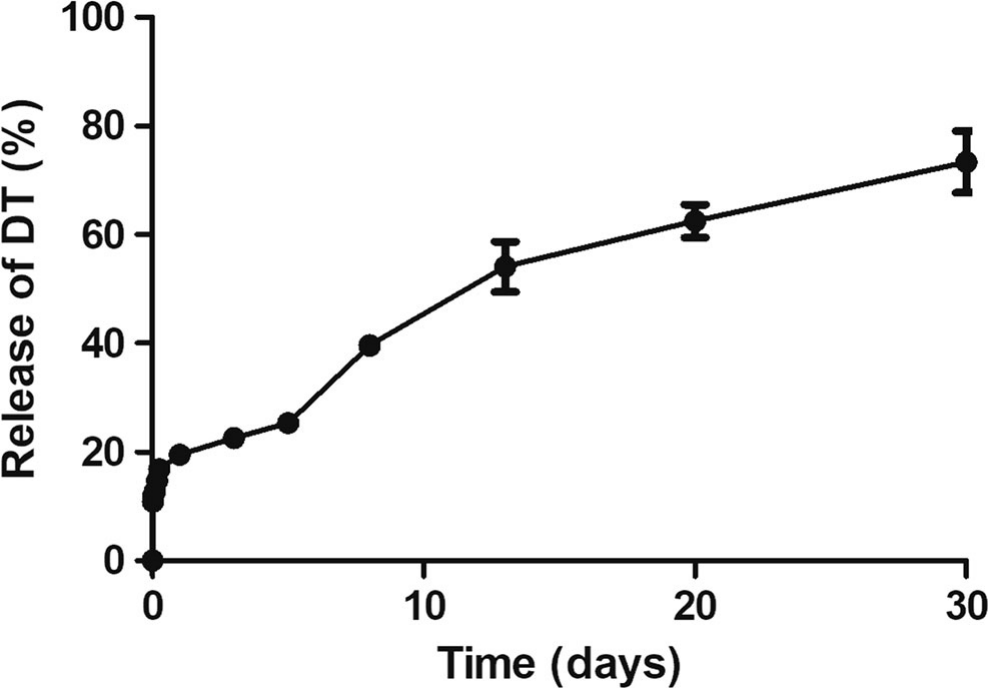
*In vitro* release of DT from LB-MSN-DT in PBS at 37°C as a function of time. Bars represent mean ± SEM, *n* = 3.

The *in vitro* release of DT was investigated by suspending LB-MSN-DT in PBS for one month. As shown in Fig. 1, there was a moderate burst release of DT of about 20% within the first day, followed by a sustained release, reaching a total release percentage of about 70% on day 30. These results indicate that the LB-MSN-DT may serve as a reservoir and allow the sustained release of DT, but at the same time retain sufficient DT for a prolonged period of time to deliver it as nanoparticulate antigen to APCs.

### Quantification of Coated Amount of Nano-Encapsulated DT on Microneedle Arrays

**Fig. 2:**
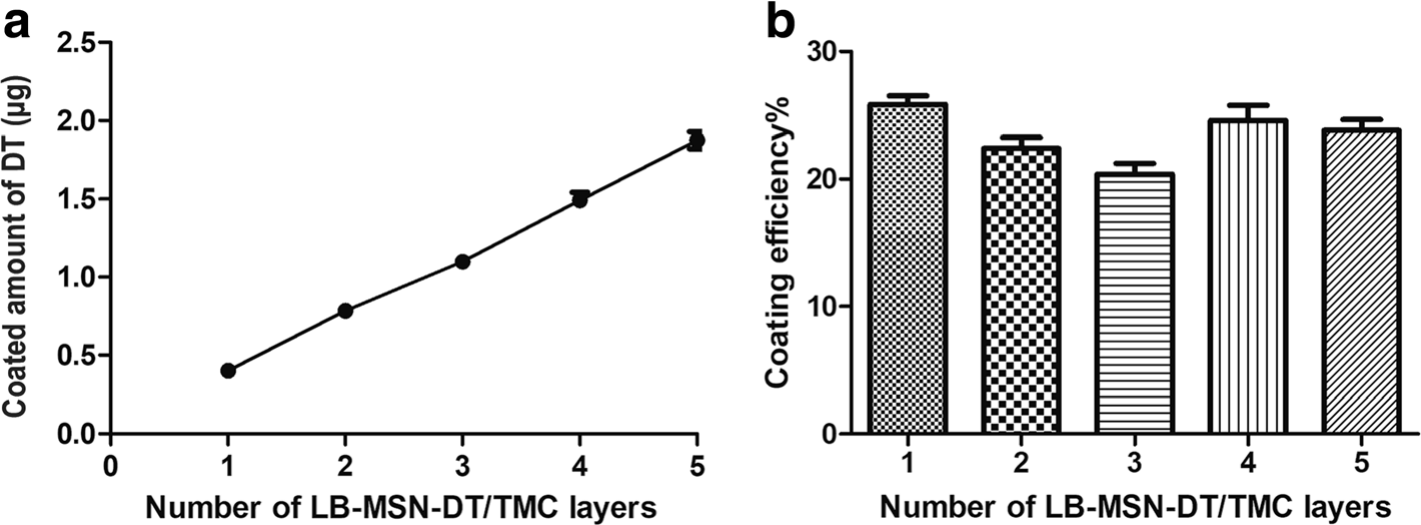
Cumulative amount of nano-encapsulated DT (**a**) that was coated on the microneedles of one microneedle array and coating efficiency (**b**) as a function of the number of layers. Data is represented as average ± SEM of 3 independent microneedle arrays.

As shown in Fig. 2a, the amount of nano-encapsulated DT that was coated onto the microneedles increased linearly with increasing number of coating layers. About 0.4 μg DT was coated onto the microneedles of one microneedle array per layer. The coating efficiency was similar for each layer and was about 20–26% (Fig. 2b). As shown in Table II, the cumulative amount of nano-encapsulated DT coated on the microneedle surfaces of one microneedle array was about 1.9 μg and 1.1 μg, corresponding to 9.7 μg and 5.7 μg LB-MSN-DT (based on the mass of MSNs) for a 5-layer and 3-layer coating, respectively.

**Table II:** Coated and Released Amount of DT/LB-MSN-DT from the Microneedles of a Single Microneedle Array (*n* = 3).

Microneedles	Coated DT (μg)	^a^Coated LB-MSN-DT (μg)	^b^Delivered DT (μg)		^c^Delivered percentage (%)	
			Multiple insertion mode	Single insertion mode	Multiple insertion mode	Single insertion mode
5-layer coated	1.9 ± 0.1	9.7 ± 0.2	0.814 ± 0.008	0.341 ± 0.083	42.8 ± 0.1%	17.9 ± 0.8%
3-layer coated	1.1 ± 0.1	5.7 ± 0.2	0.256 ± 0.001	–	23.2 ± 0.0%	–

Data are average ± SEM of 3 independent microneedle arrays

^a^The coated amount of LB-MSN-DT is expressed as the mass of MSNs and was calculated by using the coated amount of DT and loading capacity of DT in LB-MSN-DT

^b^The delivered dose of DT was measured in *ex vivo* human skin

^c^Delivered percentage was calculated by dividing the delivered amount of DT in *ex vivo* human skin by the coated amount of DT on the microneedles

### Visualization of Coated Microneedles before and after Penetrating *Ex Vivo* Human Skin by SEM

**Fig. 3:**
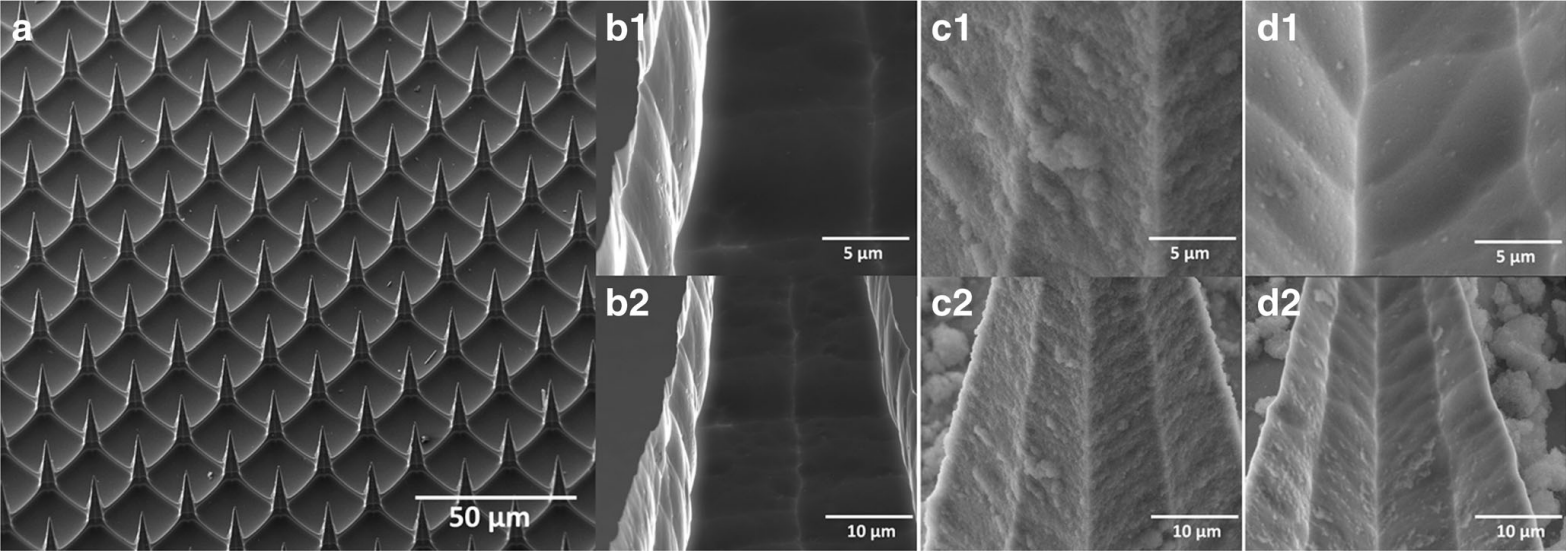
Scanning electron microscopy (SEM) images of uncoated pH-sensitive microneedles (a, b1-b2), microneedles coated with 5 layers of LB-MSN-DT/TMC (c1-c2), and the microneedles after insertion into and removal (multiple insertion mode) from *ex vivo* human skin (d1-d2).

The 5-layer LB-MSN-DT coated microneedles were visualized by SEM. The uncoated pH-sensitive microneedles showed a smooth surface (Fig. 3**a, b1-b2**). On the surface of LB-MSN-DT coated microneedles (Fig. 3 c**1-c2**), single nanoparticles or clusters of nanoparticles were observed. After insertion of the microneedles into and removal from the skin, the nanoparticle density was reduced on the microneedle surface (Fig. 3 d**1-d2**).

### Visualization of the Released LB-MSN-DT in *Ex Vivo* Human Skin

**Fig. 4:**
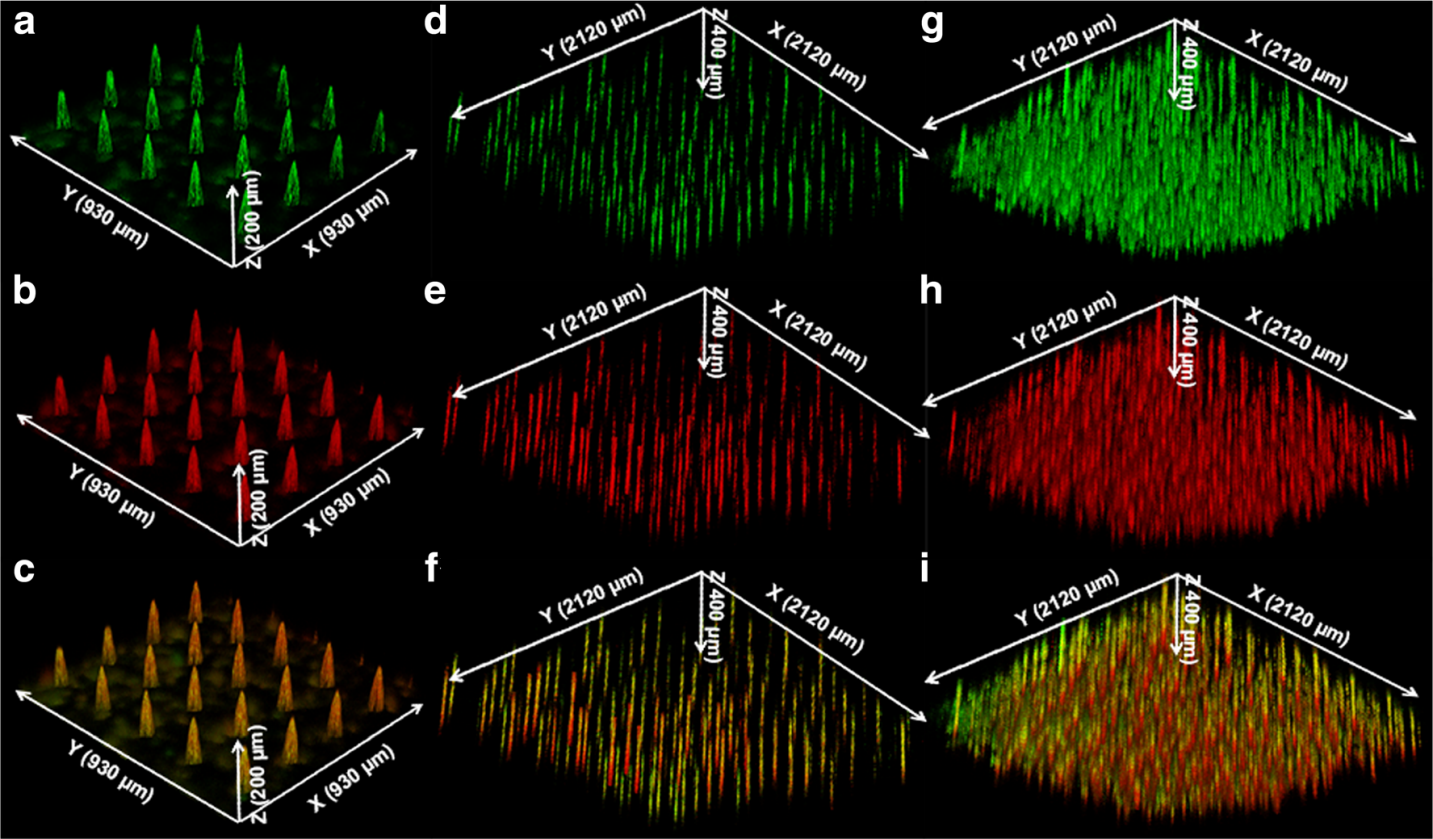
Confocal laser scanning microscopy (CLSM) images of 5-layer LB-MSN-DT coated microneedles (**a**: DT-Alexa488; **b**: TMC-Rho; **c**: merged), and *ex vivo* human skin after insertion and removal of microneedle arrays (5-layer coated) by using single (**d**: DT-Alexa488; **e**: TMC-Rho; **f**: merged) or multiple insertion mode (**g**: DT-Alexa488; **h**: TMC-Rho; **i**: merged).

After observation of the reduction of the number of nanoparticles on the microneedle surface after penetration in and withdrawal from human skin, CLSM was used to visualize the released LB-MSN-DT in the skin. To this end, the 5-layer LB-MSN-DT coated microneedles before penetration of the skin were first visualized. The green color from DT-Alexa488 (Fig. 4a) and red color from TMC-Rho (Fig. 4b) were observed and they colocalized on the surface of the microneedles (Fig. 4c). These results support the SEM images of LB-MSN-DT coated microneedles (Fig. 3 c**1-c2**), further revealing that LB-MSN-DT were successfully coated onto the surface of the microneedles.

Next, the released LB-MSN-DT in the skin was visualized by CLSM. After a single insertion, the fluorescence of the released DT-Alexa488 and TMC-Rho were clearly observed (Fig. 4d-f). The green color from DT-Alexa488 **(**Fig. 4d**)** and red color from TMC-Rho (Fig. 4e) co-localized in the micro-channels induced by the microneedles (Fig. 4f). After the microneedles were inserted in and withdrawn from the skin by using the multiple insertion mode, clearly more micro-channels were observed as indicated by the fluorescence of DT-Alexa488 and TMC-Rho (Fig. 4g-i). These results together with the SEM images of the coated microneedles after penetration of the skin indicate that LB-MSN-DT were successfully released into skin.

### Quantification of the Released Amount of DT from Microneedles into *Ex Vivo* Human Skin

As shown in Table II, after insertion of the 5-layer coated microneedle arrays into *ex vivo* human skin, the delivery efficiency from the microneedles by using the multiple insertion mode (42.8%) was more than twice as high compared to that in single insertion mode (17.9%). Based on this observation, the multiple insertion mode was chosen for subsequent penetration studies. Next, the released amounts of DT from microneedles coated with 5 and 3 layers of LB-MSN-DT were compared. The amount of delivered DT in the skin from one 5-layer coated microneedle array (0.814 μg) was about 3-fold higher than that from a 3-layer coated microneedle array (0.256 μg) (Table II).

### IgG Antibody Titers after Intradermal Vaccination

**Fig. 5:**
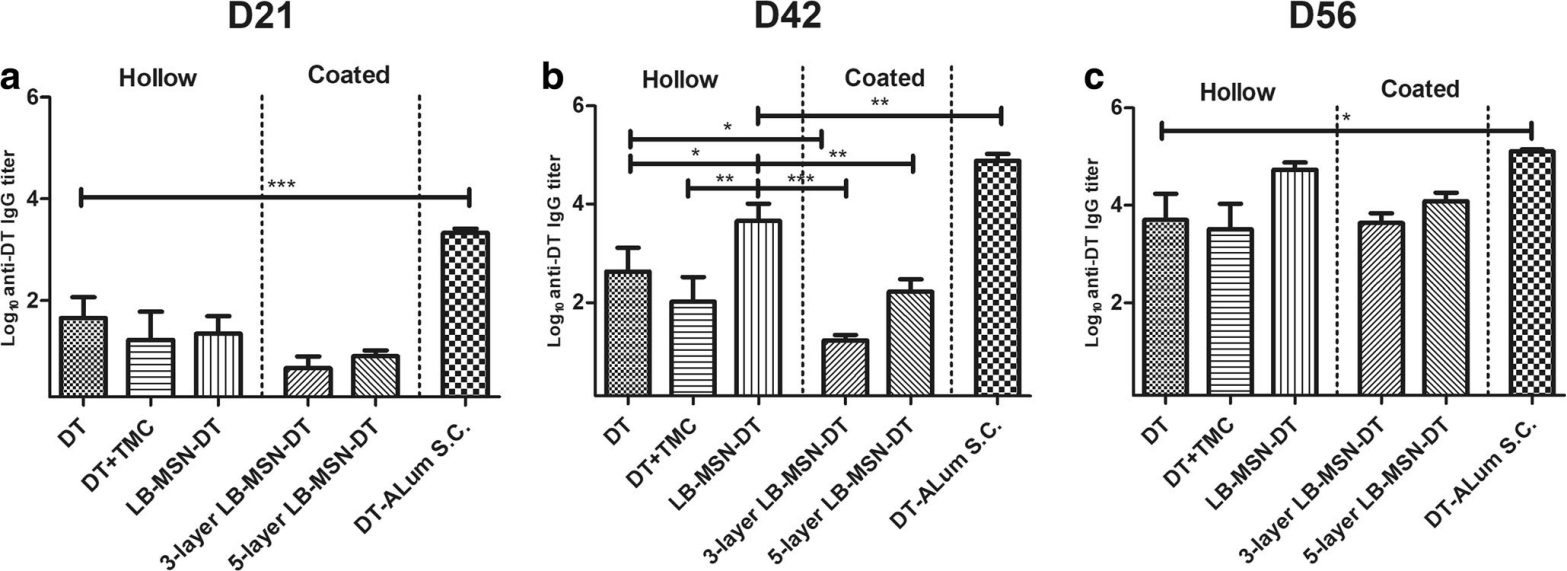
DT-specific total IgG antibody titers on day 21 (**a**), 42 (**b**) and 56 (**c**). Bars represent mean ± SEM, *n* = 8. *p < 0.05, **p < 0.01, ***p < 0.001.

Total IgG titers are shown in Fig. 5. On day 21 all groups showed detectable total IgG titers (Fig. 5a). On day 42, the responses of all groups increased compared to those on day 21. Responses of hollow microneedle injected LB-MSN-DT were significantly higher than those induced by DT/TMC solution and LB-MSN-DT coated microneedle groups (Fig. 5b) (p < 0.05). On day 21 and 42, DT-Alum induced higher total IgG responses than other groups, probably due to the much higher dose used (*p* < 0.01). On day 56, the responses of hollow microneedle injected LB-MSN-DT and 5-layer LB-MSN-DT coated microneedles increased to similar IgG levels as those induced by DT-Alum, despite the ca. 15-fold lower dose, while DT/TMC solution elicited significantly lower levels than DT-Alum.

In all these three immunizations, the addition of TMC did not improve the total IgG response. Additionally, 5-layer LB-MSN-DT coated microneedles seemed to induce a stronger total IgG response than 3-layer coated microneedles, although the difference was not significant (p˃0.05). In summary, LB-MSN-DT delivered by both coated and hollow microneedles successfully induced DT-specific total IgG responses. LB-MSN-DT induced superior total IgG responses as compared to DT/TMC solution when administered by hollow microneedles (after 1st boost), but not when using coated microneedles.

**Fig. 6:**
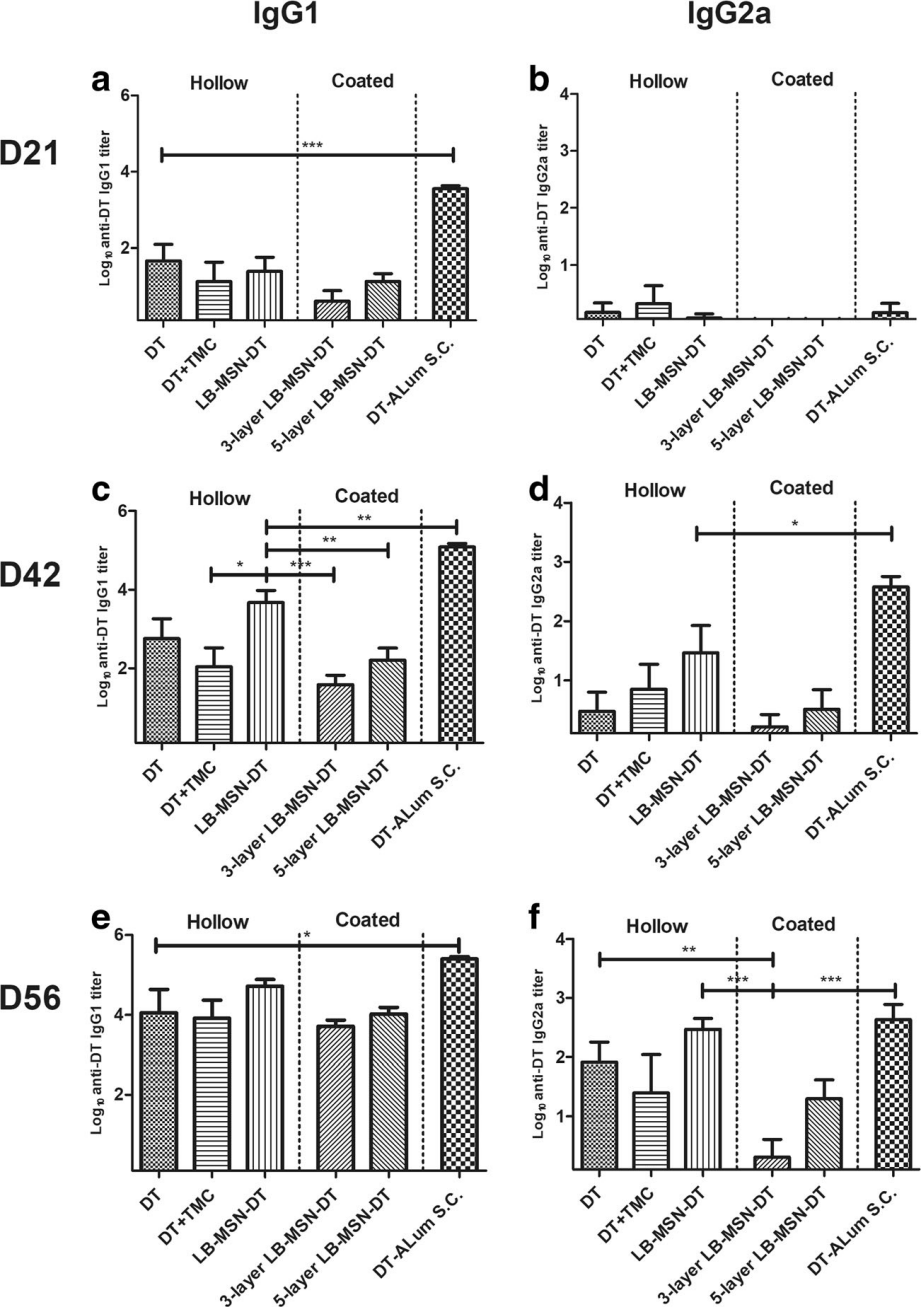
DT-specific IgG1 (**a**, **c**, **e**) and IgG2a (**b**, **d**, **f**) antibody titers on day 21 (**a**, **b**), 42 (**c**, **d**) and 56 (**e**, **f**). Bars represent mean ± SEM, *n* = 8. *p < 0.05, **p < 0.01, *** *p* < 0.001.

Besides total IgG, we measured the subtype IgG1 and IgG2a titers. As shown in Fig. 6, IgG1 followed the trend of total IgG (Fig. 6a, c, e). Hollow microneedle injected LB-MSN-DT induced stronger responses than DT/TMC solution (after 1st boost). However, this advantage of using LB-MSN-DT disappeared when LB-MSN-DT were delivered by coated microneedles. In case of IgG2a titers, on day 21 all groups except coated microneedles induced detectable IgG2a titers (Fig. 6b). On day 42, DT-Alum induced significantly higher titers than other groups (Fig. 6d) (p˂0.05). Although not significant, hollow microneedle injected LB-MSN-DT seemed to induce a higher IgG2a response compared to DT solution (*p* = 0.10) and coated microneedles (*p* = 0.12). On day 56, hollow microneedle injected LB-MSN-DT and DT solution showed significantly higher IgG2a titers than 3-layer LB-MSN-DT coated microneedle group (p˂0.01), but this was not significant compared to 5-layer LB-MSN-DT coated microneedles (Fig. 6f) (*p* = 0.15). Furthermore, the IgG2a titers induced by hollow microneedle injected LB-MSN-DT reached a level similar to those induced by DT-Alum. In summary, hollow microneedle injected LB-MSN-DT induced stronger IgG1 and IgG2a titers than LB-MSN-DT coated microneedles.

**Fig. 7:**
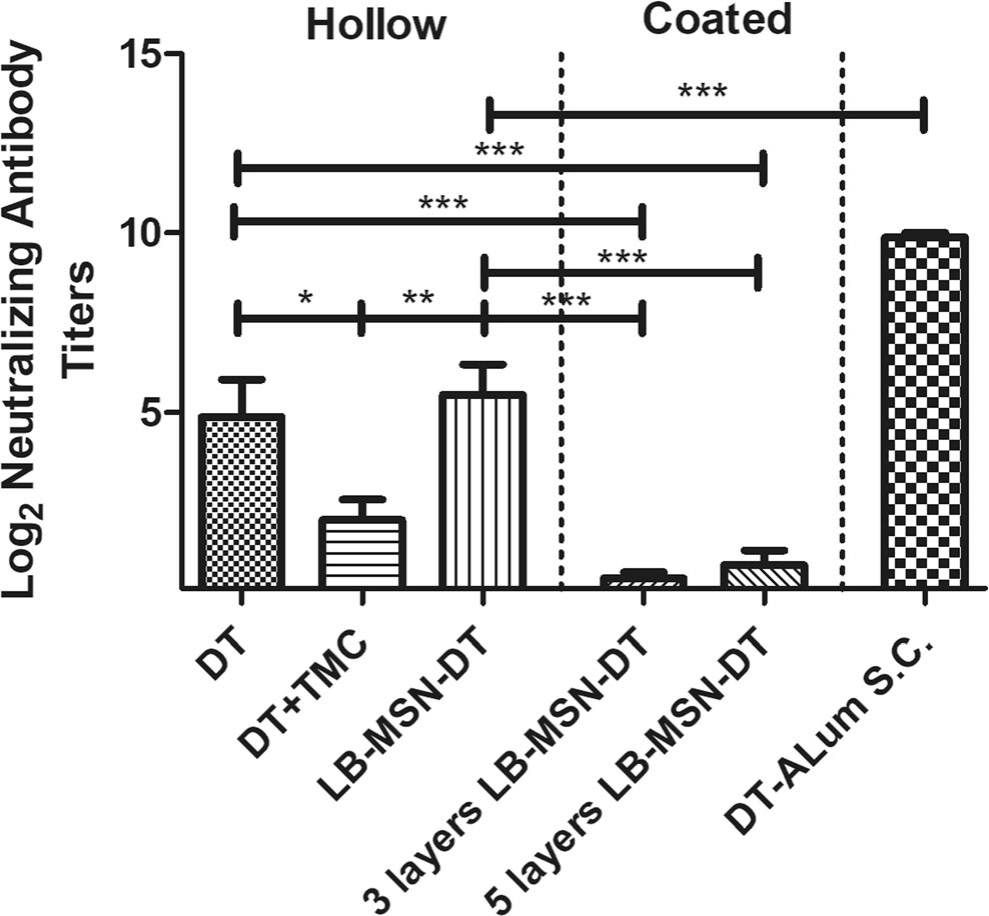
DT-neutralizing antibody titers of mice. Results are shown for serum collected on day 56. Bars represent mean ± SEM, *n* = 8. *p < 0.05, *** *p* < 0.001.

The functionality of the antibody response was determined by measuring the DT-neutralizing antibodies from serum taken on day 56. As expected, the subcutaneously injected DT-Alum with a high dose induced high neutralizing antibody titers (Fig. 7**)**. Hollow microneedle-injected LB-MSN-DT showed a significant higher neutralizing response than a mixture of DT and TMC solution and coated microneedle groups.

## Discussion

Microneedle technologies for the intradermal delivery of drugs, including vaccines, have been extensively investigated during the past twenty years (32). As the skin contains a large number of APCs, such as epidermal Langerhans cells and dermal dendritic cells, microneedles have gained particular attention as attractive delivery systems for intradermal vaccination (33). In this study, we investigated the immunogenicity of DT encapsulated MSNs after coated microneedle- and hollow microneedle-mediated intradermal immunization in mice. We showed that LB-MSN-DT delivered by both coated and hollow microneedles induced DT-specific antibody titers. Both the nano-encapsulation of DT and the type of microneedles were found to affect the immune responses.

Nanoparticulate vaccines have been reported to enhance the immunogenicity of antigens by increasing their uptake by APCs (3,34). In this study, MSNs were chosen for the loading of DT as they have large pores which allow for efficient loading of antigen (11). In a previous study it was shown that ovalbumin (OVA) loaded MSNs were able to elicit antibody responses with a reduced antigen dose compared to OVA solution adjuvanted with QuilA (9). In another study, MSNs loaded with a virus related antigen induced 10-fold higher antibody responses than the mixture of the antigen and an immune modulator (10). Our findings are in line with these results, as we showed that hollow microneedle injected LB-MSN-DT induced distinctly higher total IgG and IgG1 titers as compared to a solution of plain DT.

When coating LB-MSN-DT onto the microneedle arrays, the coated amount of DT per layer on one microneedle array (about 500 ng) was higher than that reported in a previous study (about 300 ng) where plain DT was coated onto the same type of microneedle arrays (23). The high loading capacity of DT in LB-MSN-DT together with the high surface charge of LB-MSN-DT may synergistically lead to this higher coating amount. Additionally, the multilayer coating approach used in the current study can further increase the coated amount of antigen by increasing the number of coating layers. By adjusting the number of coating layers, the coated amount of nanoparticles/antigen can be tailored.

Besides the successful coating of antigen on microneedles, it is important to have a fast release of the coating after the microneedles were penetrated into skin. Here we showed that by using a multiple insertion mode (10 penetrations within 10 s), the released amount of antigen was increased by 2.5-fold as compared to a single insertion mode. The amount of DT released into the skin was also increased compared to that released from the 5 layer coatings of plain DT using a single penetration (23). Therefore, the combination of multiple insertions with nanoparticle coatings may require less coating layers, which will facilitate the production process of coated microneedles. When using multiple insertions, the application time was much shorter than that used in single penetration mode. The improvement of release efficiency may be due to the friction force between the microneedles and the skin tissue when the microneedles were inserted in and removed from the skin. The short wearing time of microneedles by using the multiple insertion mode might help improving the acceptance by vaccinees. Nevertheless, the drawback of using the multiple insertion mode is that a sophisticated applicator needs to be developed. In the multilayer coating approach described in the current study, the TMC used has strong adhesion properties and could prevent the coated nanoparticles releasing from the microneedles (35). It would therefore be interesting to examine polymers which have weaker electrostatic interactions with the microneedles.

While hollow microneedle injected LB-MSN-DT induced a stronger immune response as compared to plain DT, LB-MSN-DT delivered by coated microneedles induced a comparable response as DT/TMC solution. The results of coated microneedles are in contrast with those reported in a recent study, which showed that nanoparticulate vaccine coated microneedles induced superior immune responses as compared to antigen solution intradermally delivered by a hypodermic needle (36). One explanation is that the dose delivered by coated microneedles may be lower than that delivered by hollow microneedles in mice skin *in vivo*. However, there are at least two arguments against this hypothesis. Firstly, our results showed that the coated microneedles delivered a two-fold higher (5-layer coated) or comparable (3-layer coated) dose in *ex vivo* human skin, respectively, compared to that delivered by hollow microneedles in human skin. The stratum corneum, viable epidermis and dermis of mouse skin are much thinner than that of human skin (37). However, the trigger to release the coating from the microneedles is the environmental pH. In the epidermis and dermis in mouse and human skin, the pH is 7.4. Therefore, the difference of the skin thickness between mouse and human skin is not expected to change the delivery efficiency. Secondly, a previous study showed that the delivered amount of DT from 5-layer coated microneedles into *ex vivo* human skin was similar as that delivered in *ex vivo* mouse skin (23). To summarize, the lower than expected responses of coated microneedle delivered LB-MSN-DT was not likely caused by lower dose of DT delivered.

Although the LB-MSN-DT coated microneedles induced similar total IgG and IgG1 responses as compared to hollow microneedle injected LB-MSN-DT on day 56 (*p* > 0.05), they induced distinctly lower IgG2a responses. At the same time, it has been reported that nano-encapsulation of antigen can increase IgG2a responses (24,38). These results suggest that the advantage of using nanoparticles is abrogated when they are delivered by coated microneedles. One possible explanation for the lower response induced by coated microneedles is that the nanoparticles were not released from the nanoparticle/TMC layers after their deposition in the skin. As a result, the nanoparticles may be not efficiently taken up by APCs or drained to lymph nodes. In the hollow microneedle groups, the addition of TMC did not improve the immune responses either. An adjuvant effect of TMC has been reported for hypodermic needle-mediated intradermal vaccination (39). This inconsistency may be caused by the much lower dose of TMC used in our study.

Previous studies have shown that IgG1 titers may be mainly responsible for the neutralizing titers against diphtheria toxin (31). However, our results showed that although hollow microneedle and coated microneedle groups induced IgG1 responses close to those induced by DT-Alum, they still induced much lower neutralizing antibodies. These results indicate that the IgG1 titers may need to reach a certain threshold in order to achieve protection against diphtheria toxin.

## Conclusion

In this study, we showed that DT loaded MSNs can be successfully delivered into mice by using coated and hollow microneedles, and evoke DT specific antibody responses. When inserting coated microneedles into skin, the multiple insertion mode of the applicator significantly increased the release efficiency of the coating compared to the single insertion mode. DT encapsulated in MSNs induced a stronger antibody response than antigen solution when delivered by hollow microneedles (after 1st boost), but not by coated microneedles. Our results revealed that both the nano-encapsulation of DT and the type of microneedles affect the immunogenicity of the antigen.

### ACKNOWLEDGEMENTS AND DISCLOSURES

We thank Hilde Vrieling and Amy Kogelman from Intravacc for their help with the neutralizing antibody assay. Guangsheng Du acknowledges the support from China Scholarship Council.

## Abbreviations

APCs: Antigen-presenting cells
CLSM: Confocal laser scanning microscopy
DLS: Dynamic light scattering
DOPC: 1,2-dioleoyl-sn-glycero-3-phosphocholine
DOPS: 1,2-dioleoyl-sn-glycero-3-[phospho-L-serine](sodium salt)
DT: Diphtheria toxoid
EE: Encapsulation efficiency
LB-MSN-DT: DT loaded and lipid fused MSNs
LC: Loading capacity
MSNs: Mesoporous silica nanoparticles
PB: Phosphate buffer
PBS: Phosphate buffered saline
PDI: Polydispersity index
SEM: Scanning electronic microscopy
TMB: 3,3′,5,5′-tetramethylbenzidine
TMC: N-trimethyl chitosan
